# PDK1 elevation was induced by epigenetic modifications of KDM3A and METTL16 to mediate TKI resistance and cancer development

**DOI:** 10.1016/j.gendis.2025.101947

**Published:** 2025-11-25

**Authors:** Zhihao Zhou, Ruike Zhang, Zhaoyang Zhang, Liyuan Zhang, Wei Wang, Wenjing Liu, Chunyang Zhang, Gen Lin, Weimiao Yu, Bo Xu, Lin Wang, Bing-Hua Jiang

**Affiliations:** a Tianjian Laboratory of Advanced Biomedical Sciences, Academy of Medical Science, Zhengzhou University, Zhengzhou, Henan 450000, China; b The Third Affiliated Hospital of Zhengzhou University, Tianjian Laboratory of Advanced Biomedical Sciences, Zhengzhou University, Zhengzhou, Henan 450052, China; c The Affiliated Cancer Hospital of Zhengzhou University, Zhengzhou, Henan 450000, China; d School of Chemistry and Chemical Engineering, State Key Laboratory of Digital Medical Engineering, Southeast University, Nanjing, Jiangsu 211189, China; e Cancer Center, Beijing Chest Hospital, Capital Medical University & Beijing Tuberculosis and Thoracic Tumor Research Institute, Beijing 101149, China; f Bioinformatics Institute, A∗STAR 138671, Singapore; g Institute of Molecular and Cell Biology, A∗STAR 138673, Singapore; h Chongqing Key Laboratory of Intelligent Oncology for Breast Cancer, Intelligent Oncology Innovation Center Designated by the Ministry of Education, Chongqing University Cancer Hospital and Chongqing University School of Medicine, Chongqing 400030, China

**Keywords:** KDM3A, METTL16, PDK1, Prognostic biomarkers, TKI resistance, Tumorigenesis

## Abstract

Lung cancer is the leading cause of cancer-related death and has the second-highest incidence worldwide. For patients with advanced EGFR-mutated non-small cell lung cancer, EGFR tyrosine kinase inhibitors (EGFR-TKIs) are the preferred treatment option; however, acquired resistance to TKIs is inevitable. Gefitinib and osimertinib, the first-generation and third-generation EGFR-TKI, have shown promising results in patients with EGFR-mutated lung cancer in clinical treatment. Here, we identified that pyruvate dehydrogenase kinase 1 (PDK1) was up-regulated in gefitinib- and osimertinib-resistant cell lines, and PDK1 knockdown rendered cells more sensitive to TKI treatment. PDK1 expression levels were significantly increased in lung, colon, liver, and breast cancer tissues compared with those in normal tissues. Histone demethylase KDM3A was also induced in TKI-resistant cell lines, and demethylated histone H3 lysine 9 to facilitate PDK1 expression to regulate TKI resistance. Further study demonstrated that METTL16 promoted the m^6^A modification of PDK1 mRNA, and the m^6^A reader IGF2BP1 directly recognized and enhanced PDK1 mRNA stability. Interestingly, KDM3A also induced METTL16 expression. Moreover, PDK1 inhibitor JX06 rendered cancer cells more sensitive to gefitinib treatment *in vivo*, and JX06 and gefitinib combination treatments have a synergic effect to inhibit tumor growth. In conclusion, the KDM3A/METTL16/PDK1 axis plays an important role in cancer development and TKI resistance, which may offer new prognostic biomarkers and therapeutic targets for TKI resistance in the future.

## Introduction

Lung cancer is one of the most common and fatal malignancies worldwide.^1^ Non-small-cell lung cancer (NSCLC) accounts for 80%–85% of all lung cancers.^2^ Recent breakthroughs in early detection and therapy allow for a more targeted strategy for treating lung cancer. In the clinical real world, therapy that targets the individual oncogenic driver mutation can restrict tumor development and provide a positive prognosis.^3^^,^^4^ Activating mutations of the epidermal growth factor receptor (EGFR) in NSCLC are an effective predictor of EGFR tyrosine kinase inhibitor (TKI) treatment.^5^^,^^6^ The standard first-line treatment for lung cancer patients with EGFR-exon 19 deletions or exon 21 L858R mutations is gefitinib.^7^ Osimertinib is the third-generation of EGFR-TKIs. EGFR TKIs enhance response rate, time to progression, and overall survival. Nevertheless, most patients will develop acquired resistance after 10–24 months of gefitinib or osimertinib treatment; the precise mechanism is unknown.^8^ As a result, it is essential to investigate the molecular mechanisms of TKI acquired resistance as well as strategies for reversing TKI resistance in lung cancer.

Pyruvate dehydrogenase kinase 1 (PDK1), a major glycolysis gene, is a member of the mitochondrial protein kinase family and is overexpressed in a wide range of malignancies, which is associated with a poor prognosis and survival.^9^, ^10^, ^11^ Studies have reported that PDK1 promotes tumor growth and facilitates aerobic glycolysis (the Warburg effect) and is involved in drug resistance in NSCLC.^12^ However, the precise molecular mechanisms underlying PDK1 overexpression specifically in EGFR-TKI-resistant NSCLC cells remain to be elucidated. To address this knowledge gap, this study systematically investigated the molecular basis for the aberrantly elevated PDK1 expression observed in EGFR-TKI-resistant cell models, employing a comprehensive approach to investigate its gene expression. Our investigation encompassed potential dysregulations at the transcriptional and post-transcriptional levels.

The methylation process plays an essential role in epigenetically associated gene expression patterns in cancer.^13^ Histone methylation has been established to be important in embryonic biology, neurological diseases, and several cancer types.^14^^,^^15^ KDM3A, which belongs to histone H3 lysine 9 (H3K9) demethylases, was demonstrated to promote tumorigenesis.^16^, ^17^, ^18^ KDM3A was discovered in this study to modulate PDK1 levels; however, the mechanism remains to be investigated. RNA N6-methyladenosine (m^6^A) modification exists in approximately 25% of mRNAs throughout the transcriptome.^19^ RNA m^6^A is modulated by the writers RNA m^6^A methyltransferases (METTL3, METTL14, and METTL16), demethylases (FTO and ALKBH5), and binding proteins (YTHDC1-2, YTHDF1-3, and IGF2BP1-3).^20^ These RNA m^6^A modification proteins are commonly exacerbated or suppressed in human cancer tissues, and they have been associated with a poor prognosis.^21^^,^^22^ Interestingly, we found that METTL16 levels were increased in TKI-resistant cells and influenced PDK1 levels; however, the mechanism is unclear, which needs to be elucidated.

In this study, lung cancer cell lines PC-9 and HCC827 with an EGFR gene mutation and sensitive to gefitinib and osimertinib were treated with gefitinib or osimertinib for six months to obtain gefitinib and osimertinib (TKI) resistant cell lines. By comparing the functions of TKI-sensitive cells (PC-9) and gefitinib-resistant cells (PC-9/G), we found that the gefitinib-resistant cells had stronger glycolytic ability. In this study, we elucidated the following four scientific questions: i) what role PDK1 up-regulation has in the acquired resistance to TKI treatment and cancer development; ii) whether histone demethylation regulates PDK1 expression during the acquired resistance of lung cancer cells to TKIs; iii) whether RNA m^6^A methylation regulates PDK1 gene expression during the TKI acquired resistance; iv) whether small molecule PDK1 inhibitor can ameliorate the up-regulation of PDK1-induced TKI resistance. The study was to elucidate new molecular mechanisms of TKI resistance through PDK1 and its upstream mediators and downstream effectors for overcoming EGFR-TKI resistance.

## Materials and methods

### Cell culture and reagents

The NSCLC cell lines PC-9 and HCC827 were purchased from the American Type Culture Collection. Gefitinib-resistant PC-9/G cell line was constructed through long-term treatment with gefitinib in PC-9 cells. Cells were cultured in RPMI 1640 medium supplemented with 10% fetal bovine serum at 37 °C with a humidified atmosphere of 5% CO_2_. Gefitinib and JX06 were purchased from MedChemExpress (Shanghai, China). PCR primers and shRNAs in this study were custom-synthesized as shown in Tables S1 and S3. The primary antibodies against KDM3A, PDK1, β-Actin, METTL16, Histone H3, H3K9me1, H3K9me2, IGF2BP1, and horseradish peroxidase-conjugated secondary antibodies were listed in Table S2.

### Proliferation, migration, and invasion

The cell proliferative capacity was measured using the cell counting kit-8 assay (Vazyme, Nanjing, China) according to the manufacturer’s manual. Concisely, cells were seeded into 96-well plates and incubated at 37 °C. The measurements were taken after 24 h, 48 h, 72 h, 96 h, and 120 h, and signals were collected by a microplate reader with OD 450 nm. For the cell migration assay, 50,000 cells were seeded into each upper chamber in 150 μL serum-free medium, and 600 μL 10% fetal bovine serum-containing medium was added to the lower chamber. After incubation for 18–24 h, cells were stained with crystal violet and methanol solution for 15 min. Cells that attached to the upper side of the chamber were removed with cotton swabs. Cells on the underside of the chamber were examined by a light microscope. For the cell migration assay, Matrigel was mixed with serum-free RPMI 1640 medium in a 1:6 ratio; 60 μL of the mixture was added to each upper chamber and incubated at 37 °C for 3 h to facilitate coagulation. Subsequently, 8 × 10^5^ cells were seeded into each upper chamber in 150 μL serum-free RPMI 1640 medium, and 600 μL 10% fetal bovine serum-containing medium was added to the lower chamber. The remaining protocol was consistent with cell migration.

### Western blotting

The cell lysates were extracted in an ice-cold lysis buffer containing phenylmethylsulfonyl fluoride (PMSF) inhibitor (Beyotime, Jiangsu, China). Protein samples were separated by electrophoresis in 8% sodium lauryl sulfate polyacrylamide gel and then transferred to the PVDF membrane (Thermo Scientific, Waltham, Massachusetts, USA), followed by blocking the membrane with 5% skim milk for 2 h and incubation with specific primary antibody at 4 °C overnight. The Imaging System was used to scan immunoblots with enhanced chemiluminescence substrates (Thermo Scientific, Waltham, Massachusetts, USA), following incubation with the related horseradish peroxidase-conjugated secondary antibody.

### Quantitative real-time PCR

Total RNAs were extracted from the indicated cells using TRIzol reagent (Invitrogen, Waltham, Massachusetts, USA) according to the manufacturer’s manual, and complementary DNA was synthesized by reverse transcription of 1 μg mRNA using HiScript III ALL-in-one RT SuperMix Perfect for quantitative PCR (Vazyme, Nanjing, China). The mRNA levels were measured by quantitative real-time PCR, and β-actin served as the internal control.

### RNA stability assay

Cells were seeded into six-well plates and cultured in a 37 °C incubator. 5 μg/mL actinomycin D (MedChemExpress, Shanghai, China) was used to treat indicated cells for 0 h, 2 h, 4 h, 6 h, 8 h, and 16 h, followed by RNA extraction. The cDNA library was constructed using a reverse transcription kit as described above and quantified by semi-PCR. The primers for semi-PCR are presented in Table S1. The mRNA expression levels at the indicated times were calculated and normalized to β-actin control.

### m^6^A dot blotting

m^6^A dot blotting was used to measure the total level of m^6^A in different cell lines. The assay was briefly conducted as follows: High concentrations of RNAs were collected using TRIzol, RNAs were incubated at 65 °C for 5 min and put on ice quickly, and then RNAs were added to nitrocellulose membrane A for the m^6^A test with membrane B serving as the control. Ultraviolet light cross-linker was used to cross-link RNA for 90 s. Membrane B was washed and incubated with 0.04% methylene blue for 5 min, followed by washing until the background of the membrane became clean. Membrane A was washed with Tris-buffered saline-Tween-20 (TBST) and incubated with 5% skim milk for 1.5 h, and then was incubated with m^6^A antibody at 4 °C overnight, followed by washing with TBST and incubation with the second antibody for 2 h. Finally, the membrane was washed with TBST and prepared for exposure.

### Luciferase reporter assay

The PDK1 wild-type sequence was cloned into the pmirGLO reporter vector. For mutant reporter plasmids, cytosine (C) replaced marked adenosine (A) in the m^6^A motif. HEK293T cells were seeded into 96-well plates and then co-transfected with 0.25 μg of wild-type or mutated PDK1 reporter plasmids and 0.25 μg of pcDNA3.1-control vectors or pcDNA3.1-METTL16 plasmids. After 36 h, the luciferase activity was assessed using Duo-LiteTM Luciferase Assay system (Vazyme, Nanjing, China) according to the manufacturer’s manual.

### Apoptosis analysis

Cells were seeded in 6-well plates and treated with different drugs for 48 h. Then, cells were collected according to the instructions and stained with annexin V and propidium iodide for further flow cytometry analysis. For the TUNEL assay, cells were seeded in EZ Slide 8-Well Glass (Sigma–Aldrich, Darmstadt, Germany). After indicated treatment for 48 h, the cells were fixed with methanol for 20 min. Apoptotic cells were measured using the One-step TUNEL Cy5 Apoptosis Detection Kit (APExBIO, Houston, TX, USA) according to the manufacturer’s instructions. For the mitochondrial membrane potential assay, cell culture and drug methods were consistent with the above. After different treatments for 48 h, the mitochondrial membrane potential of cells was detected with the enhanced mitochondrial membrane potential assay kit using JC-1 probe (Beyotime, Jiangsu, China), and the fluorescence was analyzed with a fluorescence microscope.

### Tube formation assay

To prepare conditioned medium, target cells were seeded until they reached 70%–80% confluence and subjected to incubation for 48 h after the medium was replaced with serum-free RPMI 1640, followed by collecting, aliquoting, and storing the conditioned medium at −80 °C. For the tube assay, 5 × 10^5^ human umbilical vein endothelial cells were seeded in a 60 mm dish and cultured overnight to 80%–90% confluence and then serum-starved for 12 h. One day prior, growth factor-reduced Matrigel was thawed at 4 °C and pre-chilled a 96-well plate for 2 h. Before use, Matrigel was diluted with serum-free RPMI 1640 on ice at a ratio of 1:1, aliquoted 60 μL/well, and polymerized at 37 °C for 30 min. The starved human umbilical vein endothelial cells were harvested, counted, and pelleted at 8 × 10^4^ cells per group. The pellet in 400 μL of the specific conditioned medium (100 μL/well, quadruplicate) was resuspended and incubated at 37 °C/5% CO_2_ for 4 h, followed by imaging of the capillary networks and quantification of tube lengths with ImageJ.

### RNA immunoprecipitation

RNA immunoprecipitation assay was performed using a Magna RNA immunoprecipitation kit (Sigma–Aldrich, Darmstadt, Germany) according to the manufacturer’s instructions. Briefly, 5 μg anti-N6-methyladenosine (m^6^A) and anti-rabbit IgG were incubated with 50 μL magnetic beads before cell lysates were added. Then, the RNA-protein complexes were washed 6 times, and proteinase K digestion buffer was used for incubation to remove the proteins. Finally, RNAs were extracted by phenol-chloroform RNA extraction and purified for quantitative PCR analysis.

### Chromatin immunoprecipitation

Chromatin immunoprecipitation assays were performed with the SimpleChIP plus Enzymatic Chromatin IP kit (Cell Signaling Technology, Danvers, Massachusetts, USA) according to the manufacturer’s protocol. Briefly, the cells were harvested and bonded with beads. After incubation with specific antibodies or normal rabbit IgG, the chromatin was bound to beads and extracted. Then, the cell’s chromatin samples were detected by quantitative reverse transcription PCR; the primer sequences used for PCR are listed in Table S1.

### Mouse xenograft model

The 4-week-old male BALB/C nude mice were purchased from Beijing HFK Bioscience Co., Ltd., China. PC-9/G cells (5 × 10^6^ cells) were suspended in 100 μL serum-free RPMI1640 medium and subcutaneously inoculated into the flank of each mouse. After 10 days, the tumor-bearing mice with similar tumor size were randomly divided into four groups. Then, mice were treated with phosphate-buffered saline (control), JX06 (30 mg/kg, intraperitoneal injection), gefitinib (25 mg/kg, gavage administration), or gefitinib plus JX06 twice a week. The tumor volume was measured with a 2-day interval. In addition, tumor volumes were measured with calipers and calculated according to the formula: (width^2^ × length)/2. The mice were sacrificed in the end, and the tumor tissues were collected for tests, such as immunoblotting analysis and immunohistochemistry. The study was approved by the Institutional Committee on Animal Care of Henan Cancer Hospital (Ethics No. 2021-KY-0039-001).

### Statistical analysis

Data were presented as mean ± standard deviation from three independent experiments and analyzed by a two-tailed Student’s *t*-test. Differences were considered significant at a *P*-value of < 0.05.

## Results

### Glycolysis gatekeeper PDK1 was an essential regulator of TKI resistance in lung cancer

**Figure 1:**
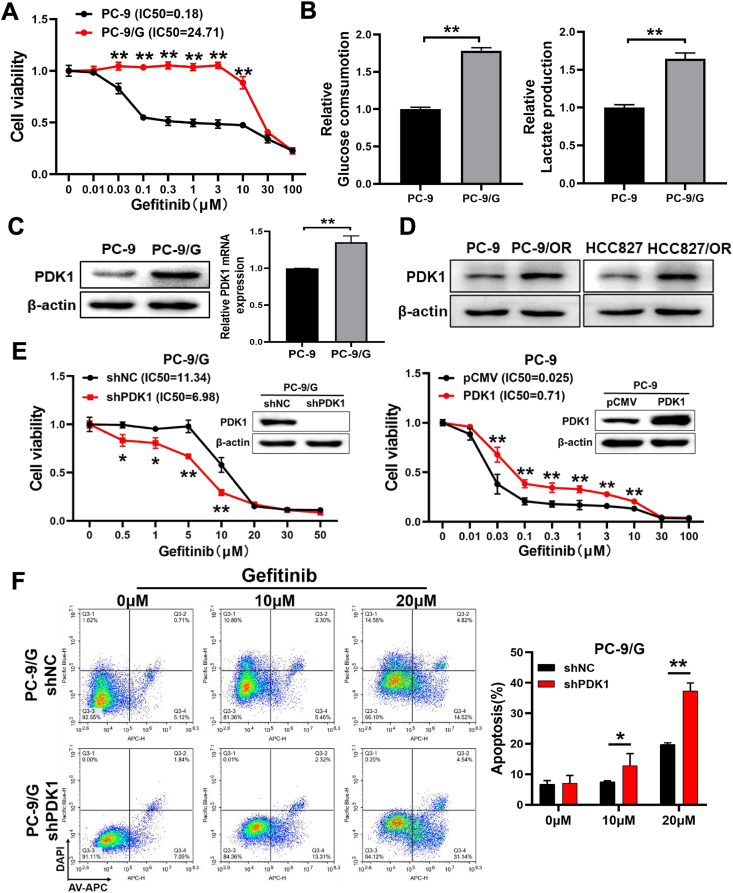
Glycolysis gatekeeper PDK1 was an essential regulator of gefitinib resistance in lung cancer. **(A)** The PC-9 and PC-9/G cells were treated with various concentrations of gefitinib for 72 h, and cell viabilities were detected by CCK-8 assay. **(B)** PC-9/G cells induced higher glucose consumption and lactate production rates compared with PC-9 cells. **(C)** The mRNA and protein expression levels of PDK1 were tested by quantitative reverse transcription PCR and Western blotting in PC-9 and PC-9/G cell lines. **(D)** The protein expression levels of PDK1 were tested by Western blotting in PC-9, PC-9/OR, HCC827, and HCC827/OR cell lines. **(E)** Indicated cells were treated with gefitinib of different concentrations for 72 h, and PDK1 knockdown in PC-9/G cells rendered cells more sensitive to gefitinib, while forced expression of PDK1 in PC-9 cells made cells more resistant to gefitinib. **(F)** The apoptosis rates were determined by flow cytometry, and PDK1 knockdown in PC-9/G cells induced higher apoptosis rates. Data were statistically analyzed with Student’s *t*-test, and values were shown as mean ± standard deviation. ∗*P* < 0.05 and ∗∗*P* < 0.01.

Acquired resistance is an obstacle to EGFR TKI therapy in lung cancer, while monitoring and eliminating EGFR TKI resistance are critical clinical challenges. A recent study has found that metabolic tumor burden, defined as total lesion glycolysis (TLG), predicts survival rate, and a high total lesion glycolysis is strongly linked with lower survival in NSCLC patients treated with first-line gefitinib.^23^ Here, the *EGFR*-mutant NSCLC cell line PC-9 was exposed to gefitinib at gradually increasing concentrations for more than 6 months to establish the gefitinib-resistant cell line PC-9/G. As shown in Figure 1A, CCK-8 analysis disclosed gefitinib sensitivity of PC-9 sensitive cells and PC-9/G resistant cells, with the IC50 values of 0.18 μM and 24.71 μM for PC-9 and PC-9/G, respectively. To further investigate the glycolysis features of PC-9 and PC-9/G cells, we firstly examined the glucose-consuming capacity and lactate-producing capacity between these two cell lines, which showed that PC-9/G cells had stronger ability of glucose consumption and lactate production compared with PC-9 cells (Fig. 1B). We then examined the expression levels of PDK1, a key regulator of glycolysis, and found that PDK1 had a higher expression level in PC-9/G cells compared with PC-9 cells (Fig. 1C). We also detected PDK1 in cells resistant to osimertinib, a third-generation EGFR-TKI, and found that PDK1 expression levels were up-regulated in two different osimertinib-resistant cell lines, HCC827/OR and PC-9/OR (Fig. 1D). We then overexpressed PDK1 in PC-9 cells and showed that PDK1 overexpression in PC-9 cells increased gefitinib resistance; whereas PDK1 knockdown in PC-9/G cells enhanced gefitinib sensitivity (Fig. 1E). We then carried out a flow-based apoptosis assay in PDK1 knockdown or control cells, and the percentage of apoptotic cells treated with gefitinib in PDK1 knockdown group was significantly increased (Fig. 1F). These results strongly suggested that PDK1 may play a crucial role in both gefitinib and osimertinib resistance.

### PDK1 promoted glycolytic metabolism and cell proliferation; higher PDK1 levels were associated with lung cancer poor prognosis and development, as well as the development of liver, colon, and breast cancer

**Figure 2:**
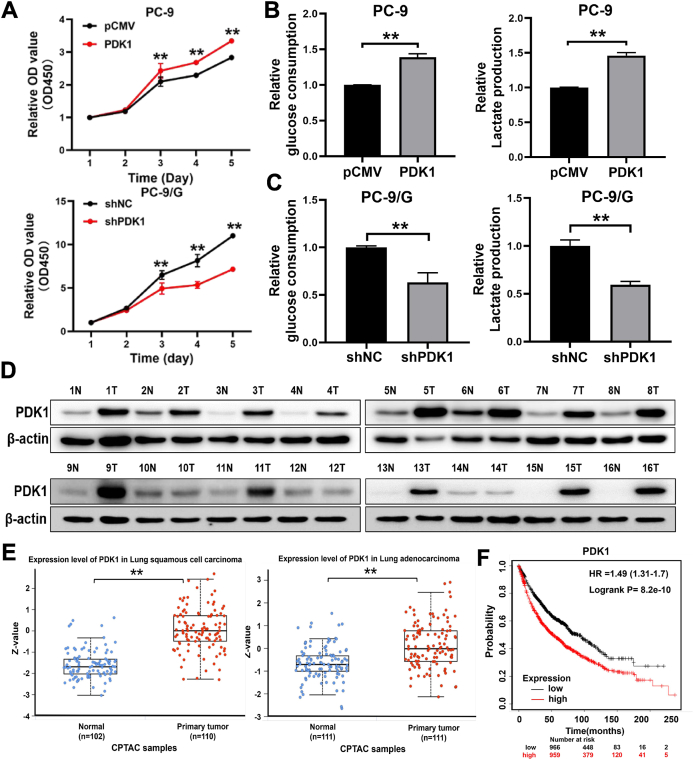
PDK1 promoted glycolytic metabolism and cell proliferation, and higher PDK1 levels indicated poor prognosis in lung cancer. **(A)** Overexpression of PDK1 significantly increased the proliferation rates of PC-9 cells, while PDK1 knockdown significantly reduced the proliferation rates of PC-9/G cells. **(B)** Overexpression of PDK1 in PC-9 cells promoted glucose consumption and lactate production activities. **(C)** Inhibition of PDK1 in PC-9/G cells attenuated glucose consumption and lactate production activities. **(D)** Western blotting was performed to analyze the expression levels of PDK1 in 16 matched pairs of normal and tumor specimens. β-actin was applied as the internal control. **(E)** Relative PDK1 protein levels in tumor and normal samples of lung adenocarcinoma and lung squamous cell carcinoma patients. **(F)** The Kaplan–Meier curves showed that higher PDK1 levels indicated a poor survival rate (log-rank *P* = 8.2e-10). Data were statistically analyzed with Student’s *t*-test, and values were shown as mean ± standard deviation. ∗∗*P* < 0.01.

To determine the function of PDK1 elevation in lung cancer, we conducted CCK-8 assay, migration assay, invasion assay, and tube formation assay. Our findings revealed that PDK1 overexpression in PC-9 cells resulted in enhanced proliferative activity, migration activity, invasion activity, and tube-formation activity. Conversely, PDK1 knockdown in PC-9/G cells diminished these activities (Fig. 2A; Fig. S1A–S1C). We then analyzed the TCGA database and found that PDK1 levels were positively correlated with carbon metabolism, pyruvate metabolism, and the glycolysis pathway in the lung cancer cohort, and higher PDK1 levels were positively correlated with the pyruvate metabolism pathway (Fig. S1D, S1E). We further examined the glucose-consuming capacity and the lactate-producing capacity, and showed that PC-9 cells acquired stronger glucose-consuming and lactate-producing capacities after overexpression of PDK1; conversely, PDK1 knockdown of PC-9/G cells showed decreased glucose consumption and lactate production capacities (Fig. 2B, C). In addition, the protein expression levels of PDK1 were found to be up-regulated in the lung cancer tumor samples compared with the normal samples (Fig. 2D). We analyzed CPTAC database and showed that PDK1 levels were significantly increased in lung adenocarcinoma and lung squamous cell carcinoma tissues compared with normal tissues (Fig. 2E). Moreover, higher PDK1 levels were associated with the poor survival rate of patients in lung cancer cohort (Fig. 2F). Similarly, we found that PDK1 elevations were associated with the development of other types of human cancers, including liver, colon, and breast cancers (Fig. S2). Thus, these results showed that PDK1 promoted glycolytic metabolism and cell proliferation capacities, and higher PDK1 levels were associated with the development of multiple types of cancer.

### Histone demethylase KDM3A induced the expression levels of PDK1 via H3K9me1 and H3K9me2 demethylation

**Figure 3:**
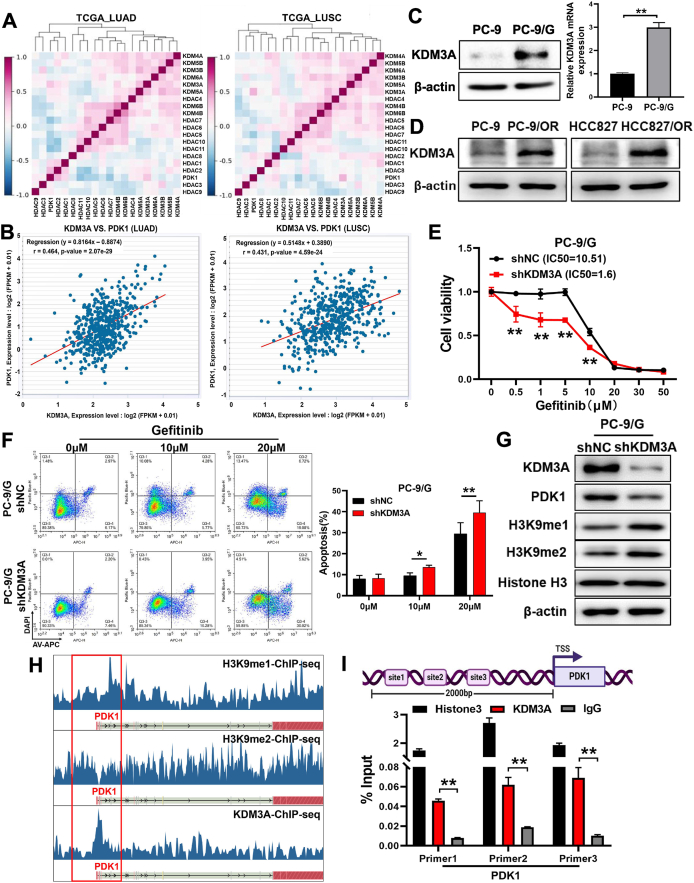
Histone demethylase KDM3A induced the expression levels of PDK1 via H3K9me1 and H3K9me2 demethylation. **(A)** The analysis results of gene correlation analysis from the LUNG CANCER EXPLORER database showed that KDM3A was positively correlated with PDK1. **(B)** The Starbase database also confirmed that there was a positive correlation between KDM3A and PDK1. **(C)** The mRNA and protein expression levels of KDM3A were tested by quantitative reverse transcription PCR and Western blotting in PC-9 and PC-9/G cells. **(D)** The protein expression levels of KDM3A were tested by Western blotting in PC-9, PC-9/OR, HCC827, and HCC827/OR cell lines. **(E)** After silencing KDM3A in PC-9/G cells, the sensitivity of the cells to gefitinib increased. **(F)** The apoptosis rates were determined by flow cytometry, and KDM3A knockdown in PC-9/G cells induced higher apoptosis rates. **(G)** KDM3A knockdown in PC-9/G cells reduced the expression levels of PDK1, while the expression levels of H3K9me1 and H3K9me2 were increased. **(H)** The ENCODE database showed there were binding peaks of H3K9me1, H3K9me2, and KDM3A at the promoter region of PDK1. **(I)** Chromatin immunoprecipitation assay was conducted to determine the binding sites of KDM3A at the promoter region of the PDK1 gene. Cross-linked and sheared chromatin was immunoprecipitated with anti-KDM3A/histone H3 antibody or IgG, and quantitative reverse transcription PCR was used to detect the enrichment percentages. Data were statistically analyzed with Student’s *t*-test, and values were shown as mean ± standard deviation. ∗*P* < 0.05 and ∗∗*P* < 0.01.

Histone methylation is the most prevalent histone modification and an effective regulator of gene transcription. Lysine demethylases regulate and maintain epigenetic variables impacting the structure of chromatin and cellular features.^24^ We performed the gene correlation analysis from LUNG CANCER EXPLORER and the Starbase database, and the results showed that KDM3A levels were positively correlated with PDK1 expression levels in lung cancer tissues (Fig. 3A, B). We found that the protein and mRNA expression levels of KDM3A were significantly increased in PC-9/G cells (Fig. 3C). In addition, the protein levels of KDM3A were significantly increased in lung cancer tissues compared with normal tissues in the CPTAC database, and higher KDM3A levels indicated a poor survival rate in lung cancer patients (Fig. S3A, S3B). Meanwhile, KDM3A expression levels were also significantly elevated in the HCC827/OR and PC-9/OR cell lines (Fig. 3D). To investigate whether KDM3A may induce drug resistance *in vitro*, we overexpressed KDM3A in PC-9 cells and silenced KDM3A expression in PC-9/G cells (Fig. S3C). KDM3A knockdown in PC-9/G cells enhanced drug sensitivity to gefitinib (Fig. 3E). We further performed apoptosis assay in KDM3A knockdown or control cells, and the percentage of apoptotic cells treated with gefitinib in KDM3A knockdown group was significantly increased (Fig. 3F). Moreover, forced expression of KDM3A promoted cell proliferation activity in PC-9 cells, and inhibition of KDM3A attenuated cell proliferation and migration activities in PC-9/G cells (Fig. S3D, S3E).

As a histone demethylase, KDM3A catalyzes the removal reaction of H3K9 mono- and di-methylation (H3K9me1 and H3K9me2), and KDM3A was reported to promote the tumorigenic potential of colorectal cancer cells through promoting WNT target gene transcription by directly erasing H3K9me2 marks.^25^ We found that the expression levels of H3K9me1 and H3K9me2 were significantly decreased in PC-9/G cells (Fig. S3F). The expression levels of PDK1 were decreased following knockdown of KDM3A in PC-9/G cells, and the levels of H3K9me1 and H3K9me2 were increased (Fig. 3G). Conversely, after overexpression of KDM3A in PC-9 cells, the expression levels of PDK1 were elevated, and the levels of H3K9me1 and H3K9me2 were decreased (Fig. S3G). KDM3A was significantly enriched in the transcriptional regulatory region of PDK1 through analysis of the Cistrome DB database (Fig. 3H). Then, we performed a chromatin immunoprecipitation assay and three pairs of quantitative PCR primers were designed to analyze the upstream promoter region of PDK1, and the results showed that KDM3A had higher enrichment in the promoter region of PDK1 gene compared with IgG (Fig. 3I). These results suggest that KDM3A may transcriptionally promoted the expression levels of PDK1 by demethylating H3K9me1 and H3K9me2 modifications.

### RNA m^6^A methyltransferase METTL16 enhanced the mRNA stability of PDK1 and induced TKI resistance in lung cancer

**Figure 4:**
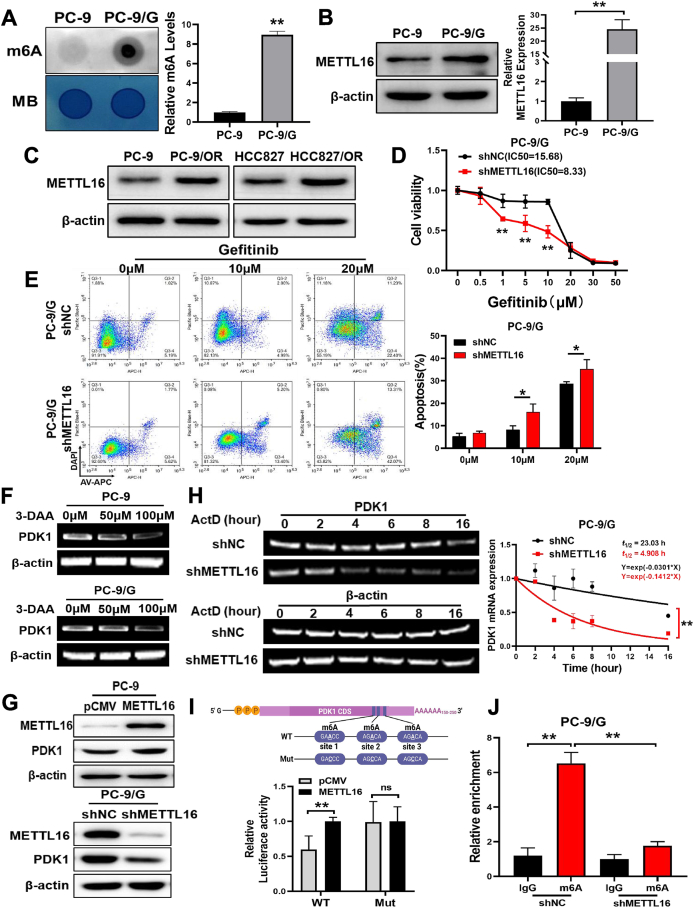
RNA m^6^A methyltransferase METTL16 enhanced the mRNA stability of PDK1 and induced gefitinib resistance in lung cancer. **(A)** The level of total M^6^A in PC-9 and PC-9/G cells was detected by dot blotting. **(B)** The mRNA and protein expression levels of METTL16 were tested by quantitative reverse transcription PCR and Western blotting in PC-9 and PC-9/G cells. **(C)** The protein expression levels of METTL16 were tested by Western blotting in PC-9, PC-9/OR, HCC827, and HCC827/OR cell lines. **(D)** After silencing METTL16 in PC-9G cells, the sensitivity to gefitinib increased. **(E)** The apoptosis rates were determined by flow cytometry, and METTL16 knockdown in PC-9/G cells induced higher apoptosis rates. **(F)** 3-DAA treatment in PC-9 and PC-9/G cells reduced the expression levels of PDK1. **(G)** Overexpression of METTL16 in PC-9 cells showed increased expression of PDK1, and knockdown of METTL16 in PC-9/G cells showed reduced expression of PDK1. **(H)** PDK1 mRNA levels were determined by semi-PCR in PC-9/G cells (control and METTL16-silenced) after actinomycin D treatment (normalized to 0 h). **(I)** Relative wild-type (WT) or mutated (Mut) luciferase activities in METTL16-overexpressed PC-9 cells were determined by normalizing the values to those of the negative control group. **(J)** The RNA immunoprecipitation assay showed that total m^6^A antibody levels were enriched on PDK1 mRNA, but were significantly decreased after METTL16 silencing. Data were statistically analyzed with Student’s *t*-test, and values were shown as mean ± standard deviation. ∗*P* < 0.05 and ∗∗*P* < 0.01.

As the most prevalent internal decoration of mammalian messenger RNA, m^6^A plays essential roles in normal biological processes and development by regulating the fate of target RNAs.^26^ In this study, the m^6^A levels in PC-9/G cells were dramatically increased compared with PC-9 cells (Fig. 4A). We further investigated the levels of several RNA m^6^A methyltransferases in PC-9 and PC-9/G cells, and found that both protein and mRNA levels of METTL16 in PC-9/G cells were greatly increased compared with PC-9 cells (Fig. 4B; Fig. S4A). Similarly, the expression levels of METTL16 were also significantly increased in the HCC827/OR and PC-9/OR cell lines (Fig. 4C). To explore the function of METTL16, we overexpressed METTL16 in PC-9 cells, and showed that forced expression of METTL16 induced m^6^A level; whereas we knocked down METTL16 in PC-9/G cells and found that METTL16 inhibition decreased m^6^A levels (Fig. S4B). Moreover, the proliferation rates of cells were increased after overexpression of METTL16, and METTL16 knockdown decreased cell growth rates (Fig. S4C). Suppression of METTL16 also inhibited the cell migration activities (Fig. S4D). Furthermore, METTL16 knockdown in PC-9/G cells enhanced drug sensitivity to gefitinib (Fig. 4D). We further performed apoptosis assay in METTL16 knockdown or control cells, and the percentages of apoptotic cells treated with gefitinib in METTL16 knockdown group were significantly increased (Fig. 4E). To explore the relationship between METTL16 and PDK1, we applied methyltransferase inhibitor 3-DAA to treat PC-9 and PC-9/G cells and found that the mRNA expression levels of PDK1 were greatly reduced (Fig. 4F). In addition, forced expression of METTL16 in PC-9 cells promoted PDK1 protein expression, whereas METTL16 suppression in PC-9/G cells decreased PDK1 levels (Fig. 4G).

It is known that METTL16 is an RNA methyltransferase that can methylate RNA. We further studied whether METTL16 could regulate the expression of PDK1 through mediating the methylation of PDK1 mRNA. We then conducted an mRNA stability assay with actinomycin D and found that PDK1 mRNA was more stable in gefitinib-resistant cells (Fig. S4E). Further research found that METTL16 overexpression in PC-9 cells prolonged the half-life of PDK1 mRNA, whereas METTL16 knockdown significantly decreased the half-life of PDK1 in PC-9/G cells through semi-quantitative PCR assay (Fig. 4H; Fig. S4F). The results indicate that METTL16 functions as an RNA methyltransferase to regulate the PDK1 expression in TKI resistance. Next, we predicted the m^6^A modification site of PDK1 through the SRAMP database (http://www.cuilab.cn/SRAMP), and selected three sites with high scores to verify the m^6^A modification sites of PDK1. We cloned a 75-base pair sequence containing these three sites from the PDK1 mRNA into the pmirGLO reporter vector. Dual-luciferase reporter assays showed that METTL16 promoted the luciferase activities of wild-type plasmids, but did not significantly affect those of mutated plasmids, suggesting that METTL16 bonded to the m^6^A modification sites of PDK1 through luciferase reporter assay (Fig. 4I). To further validate the specific m^6^A modification sites of PDK1, we performed RNA immunoprecipitation experiments in PC-9/G cells using the Magna RNA immunoprecipitation kit, and showed that m^6^A antibody was enriched with PDK1 sites, and found that METTL16 knockdown inhibited the m^6^A antibody–PDK1 binding activity in PC-9/G cells (Fig. 4J). The above results revealed that METTL16 regulated PDK1 expression through RNA m^6^A modification.

Our research demonstrated that KDM3A and METTL16 promoted PDK1 expression through transcriptional and post-transcriptional levels, respectively. Then, we tested the relationship between KDM3A and METTL16, and showed that KDM3A levels were positively correlated with METTL16 levels in the lung cancer cohort (Fig. S5A). Forced expression of KDM3A significantly promoted the expression levels of METTL16 in PC-9 cells, whereas KDM3A depletion significantly reduced METTL16 levels in PC-9/G cells (Fig. S5B, S5C). Interestingly, overexpression of KDM3A induced the m^6^A levels, whereas inhibition of KDM3A decreased the m^6^A levels (Fig. S5D). Further study showed that there was an interaction between KDM3A and METTL16 through co-IP assay (Fig. S5E, S5F). Overexpression of the KDM3A wild-type plasmid significantly increased METTL16 levels in PC-9 cells. In contrast, the KDM3A H1120Y mutant plasmid lacking demethylase activity did not affect METTL16 levels (Fig. S5G). Thus, KDM3A promoted METTL16 expression levels through the demethylation.

### IGF2BP1 stabilized PDK1 mRNA in an m^6^A-dependent manner

**Figure 5:**
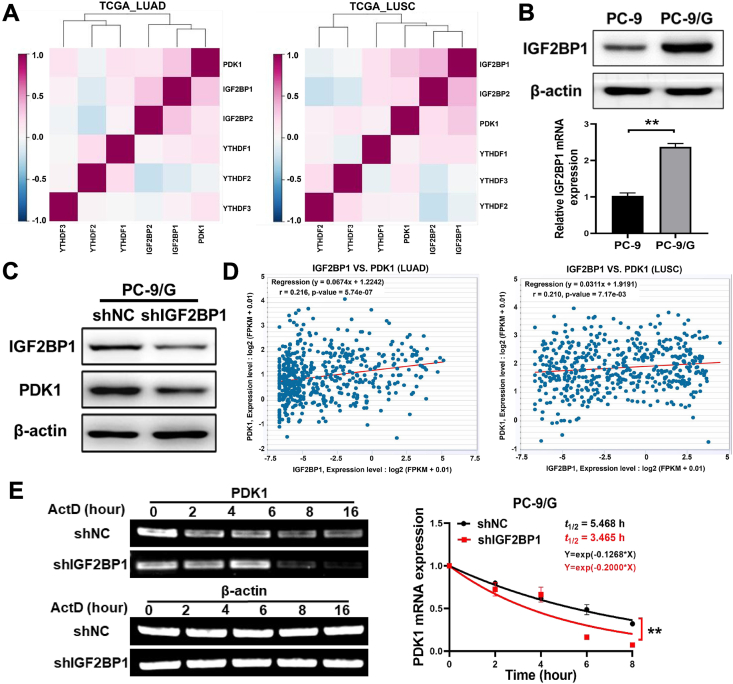
IGF2BP1 stabilized PDK1 mRNA in an m^6^A-dependent manner. **(A)** The correlation between PDK1 and m6A “reader” proteins in lung cancer cohort: IGF2BP1, IGF2BP2, YTHDF1, YTHDF2, and YTHDF3. **(B, C)** IGF2BP1 was highly expressed in PC-9/G cells, and the expression levels of PDK1 were decreased after IGF2BP1 knockdown. **(D)** The positive correlation between PDK1 and IGF2BP1 in the lung cancer cohort. **(E)** IGF2BP1 knockdown decreased the PDK1 mRNA stability. Data were statistically analyzed with Student’s *t*-test, and values were shown as mean ± standard deviation. ∗∗*P* < 0.01.

After the m^6^A modification, matured mRNAs are transported from the nucleus to the cytoplasm, where they are then identified by “readers”; the distinct readers can recognize unique m^6^A sites and are therefore implicated in RNA stabilization, translation, and degradation.^27^ Previous research has shown that IGF2BP1 may function as a reader protein and interact with particular m^6^A sites in mRNA to control cell proliferation and invasion.^28^ We performed the gene correlation analysis from LUNG CANCER EXPLORER, and showed that IGF2BP1 levels were positively correlated with PDK1 levels in the lung cancer cohort (Fig. 5A). In this research, we firstly found that the expression levels of IGF2BP1 were highly expressed in PC-9/G cells, and IGF2BP1 knockdown greatly inhibited PDK1 levels (Fig. 5B, C). We further analyzed the relationship between IGF2BP1 and PDK1 expression in the Starbase database, and showed that there was a positive correlation between IGF2BP1 and PDK1 expression levels in lung adenocarcinoma and lung squamous cell carcinoma tissues (Fig. 5D). To test whether IGF2BP1 could affect the stability of PDK1 mRNA, we performed semi-quantitative PCR experiments after treatment of PC-9/G cells with actinomycin D and found that IGF2BP1 knockdown decreased the mRNA stability of PDK1 (Fig. 5E). Thus, these results showed that IGF2BP1 acted as an m^6^A reader to regulate PDK1 expression.

### PDK1 inhibitor JX06 and gefitinib synergistically induced cell apoptosis in gefitinib-resistant lung cancer cells

**Figure 6:**
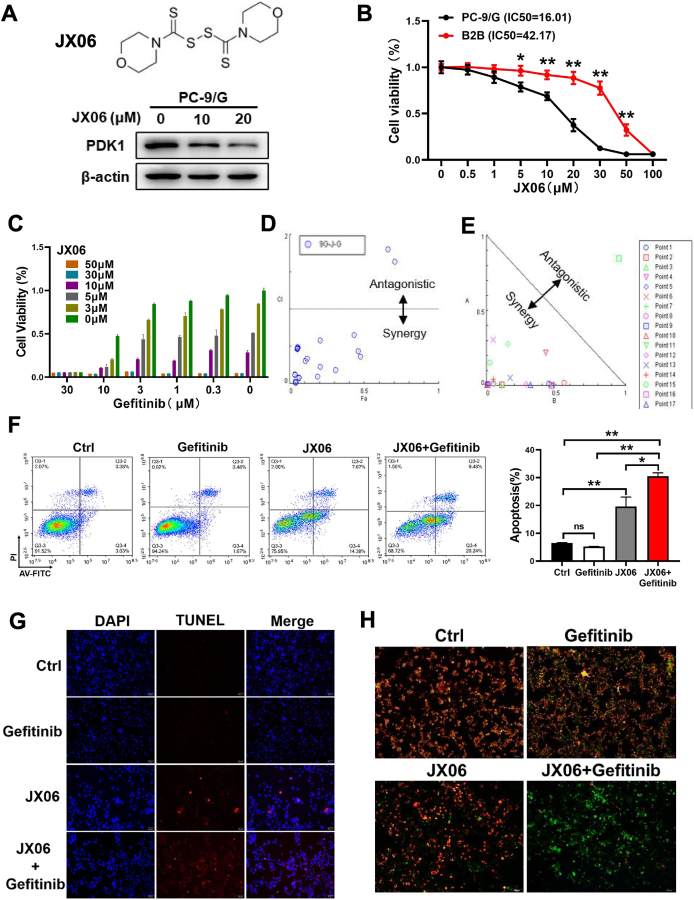
PDK1 inhibitor JX06 and gefitinib synergistically induced cell apoptosis in gefitinib-resistant lung cancer cells. **(A)** The protein expression levels of PDK were reduced upon the treatment of PDK1 inhibitor JX06 in PC-9 and PC-9/G cells. **(B)** B2B and PC-9/G cells were treated with different concentrations of JX06 for 48 h. The cell viabilities were determined by CCK-8. **(C**–**E)** The synergy effect between JX06 and gefitinib was determined and analyzed with CompuSyn software. **(F)** The apoptosis rates were analyzed with flow cytometry after the combined treatment of gefitinib and JX06. **(G)** The TUNEL assay was performed with the indicated treatment in PC-9/G cells. **(H)** The cells treated as described were stained with the JC-1 probe and detected using a fluorescence microscope. Red fluorescence indicates the aggregation form of JC-1, showing increased mitochondrial membrane potential (ΔΨm). Green fluorescence indicates the monomeric form of JC-1, which indicates reduced mitochondrial membrane potential (ΔΨm). Data were statistically analyzed with Student’s *t*-test, and values were shown as mean ± standard deviation. ∗*P* < 0.05 and ∗∗*P* < 0.01.

JX06 is a small-molecule inhibitor with a strong inhibitory effect on PDK1. It was shown that the combination of JX06-nanoparticles and metformin targeted the cancer metabolism plasticity, which significantly inhibited the growth of endometrial cancer.^29^ We treated PC-9/G cells with JX06 at different concentrations for 48 h, and found that the expression levels of PDK1 were decreased with the increasing JX06 concentrations, but PDK2 and PDK3 levels were not significantly changed (Fig. 6A; Fig. S6A). In addition, we found that PC-9/G cells were more sensitive to JX06 treatment compared with BEAS-2B cells, suggesting that JX06 is less toxic to normal cells (Fig. 6B). To investigate whether JX06 and gefitinib have a combined anti-tumor effect, PC-9/G cells were treated with JX06 (0 μM, 3 μM, 5 μM, 10 μM, 30 μM, 50 μM) and gefitinib (0 μM, 0.3 μM, 1 μM, 3 μM, 10 μM, 30 μM) and the results suggested a synergic effect between JX06 and gefitinib treatments (CI < 1; Fig. S6B), demonstrating that JX06 exerts better anti-tumor effects in combination with gefitinib (Fig. 6C–E). Furthermore, we found that JX06 in combination with gefitinib significantly increased the apoptosis rates of PC-9/G cells compared with either JX06 or gefitinib alone (Fig. 6F). Then, PC-9/G cells were treated with 1 μM gefitinib, 20 μM JX06, or gefitinib plus JX06 for 48 h, and the cells were detected with TUNEL technique to analyze apoptosis rates; the cell apoptosis rates indicated by the red fluorescence were significantly higher after dual-treatment with gefitinib and JX06 (Fig. 6G). A hallmark event early in apoptosis is the down-regulation of mitochondrial membrane potential. We examined mitochondrial membrane potential in cells treated with the indicated drugs using the enhanced mitochondrial membrane potential assay kit with JC-1. The ratios of red to green fluorescence emitted by the JC-1 fluorescent probe indicated that cells had lower mitochondrial membrane potential when treated with JX06 in combination with gefitinib, which suggested that dual-treatment with gefitinib and JX06 promoted early apoptosis in the cells (Fig. 6H). Hence, these data demonstrated that PDK1 inhibitor JX06 and gefitinib treatments synergistically induced cell apoptosis in TKI-resistant lung cancer cells.

### Dual-treatment of JX06 and gefitinib significantly inhibited tumor growth *in vivo*

**Figure 7:**
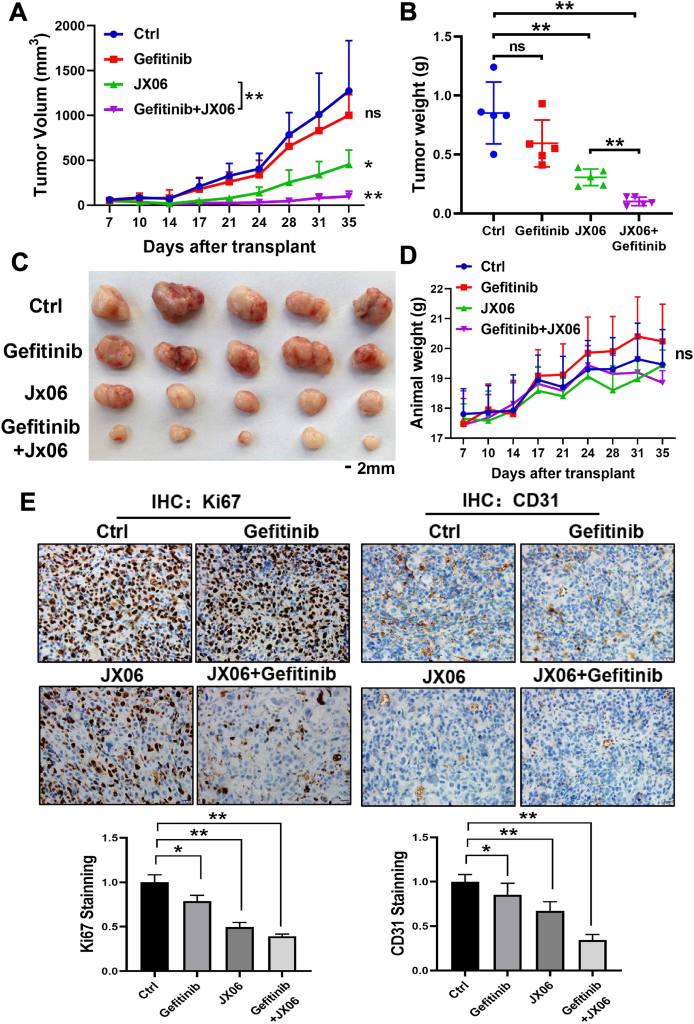
Dual-treatment of JX06 and gefitinib significantly inhibited tumor growth *in vivo*. The PC-9/G cells (5 × 10^6^ cells) were inoculated subcutaneously on the back of nude mice. When the tumor reached approximately 5 × 5 mm^2^, the nude mice were randomly divided into four groups (*n* = 5 per group) and treated with saline, gefitinib (25 mg/kg), JX06 (30 mg/kg), or gefitinib (25 mg/kg) plus JX06 (30 mg/kg). **(A**–**C)** Tumor volumes and weights were analyzed, and dual-treatment of JX06 and gefitinib significantly inhibited tumor volumes and tumor weights. **(D)** Animal weights between the four groups did not show any significant difference. **(E)** Representative images of immunohistochemical staining of Ki67 and CD31 in paraffin-embedded xenograft tumor tissues, and the expression levels of Ki67 and CD31 were quantified for six microscopic fields of the tumor samples. Data were statistically analyzed with Student’s *t*-test, and values were shown as mean ± standard deviation. ∗*P* < 0.05 and ∗∗*P* < 0.01.

To further determine the therapeutic benefit of JX06 in combination with gefitinib *in vivo*, we established a xenograft model of PC-9G cells in nude mice. The gefitinib group showed a slight inhibitory effect on tumor growth at a dose of 25 mg/kg, while co-administration of JX06 at 30 mg/kg significantly suppressed xenograft tumor growth (Fig. 7A–C). The tumors of PC-9G xenografts were weighed at the endpoint. Combination of JX06 and gefitinib showed about 70% of tumor growth inhibition, much stronger than that of JX06 or gefitinib alone (Fig. 7B). In addition, to rule out the effects of drug concentration on nude mice, the weight curves of nude mice were plotted, and there was no significant difference in weight among the four groups (Fig. 7D). Subsequently, the expression levels of Ki67 and CD31 in the four groups of tumors were detected by immunohistochemistry. The positive rates of immunohistochemistry in the combined treatment group were significantly lower than those in the other three groups. This suggests that the combination of JX06 and gefitinib can significantly inhibit tumor growth and angiogenesis (Fig. 7E). Thus, these results showed that dual-treatment of JX06 and gefitinib strongly inhibited tumor growth and angiogenesis *in vivo*.

## Discussion

EGFR-TKIs significantly improved the clinical outcomes of NSCLC patients with EGFR mutations. The first EGFR-TKI, gefitinib, was discovered to inhibit EGFR tyrosine kinase activity through binding competitively to the receptor’s intracellular ATP-binding site.^5^ Osimertinib is the third-generation of EGFR-TKIs, currently more frequently used in the clinic. Both EGFR TKIs, gefitinib and osimertinib, greatly increase response rates, time to progression, and patient overall survival. However, acquired TKI resistance inevitably develops following the treatment, leading to progression of the disease. Increased comprehension of the physiological events and molecular pathways leading to TKI resistance can help improve patient outcomes and survival rates.

Energy metabolism reprogramming, a recognized characteristic of cancer, plays a significant role in the initial development and progression of malignant tumors.^30^^,^^31^ Numerous clinical studies have shown that cancer patients with worse progression-free survival rates and a bad prognosis had elevated glycolysis levels.^32^ EGFR-mutated NSCLC cells, particularly PC-9 cells, were extremely susceptible to the EGFR-TKIs. PDK1 inhibited mitochondrial pyruvate dehydrogenase (PDH), switching glucose metabolism from mitochondrial oxidation to aerobic glycolysis. In this investigation, we evaluated the PC-9/G cell line model, which was resistant to gefitinib when compared with the parental PC-9 cells, and found that the levels of PDK1 were much higher in the PC-9/G cell line. Further research revealed that PDK1 suppression improved the response of resistant cell lines to gefitinib treatment, whereas PDK1 overexpression made sensitive cell lines resistant to gefitinib treatment. These findings suggested that PDK1 mediated TKI resistance.

Growing evidence indicates that epigenetic histone modification by lysine methyltransferases (KMTs) and demethylases (KDMs) plays an important role in gene expression modulation.^30^ A substantial number of Jumonji-C domain-containing KDMs were found to regulate a wide range of physiologic and pathological processes, including genome integrity, stem cell self-renewal, embryonic development, and oncogenesis.^14^^,^^33^ In this work, we found that KDM3A enhanced chemoresistance by removing repressive histone H3 lysine 9 methylation and activated the oncogene gene PDK1. Interestingly, KDM3A increased METTL16 expression levels, whereas KDM3A knockdown decreased m^6^A levels via METTL16 inhibition. To support the direct link, we showed that the single AA mutant of KDM3A, the demethylase-deficient KDM3A H1120Y mutant, did not affect METTL16 expression. We found that the lysine-specific demethylase KDM3A had a significant impact on lung cancer chemoresistance by demethylating histone and non-histone proteins.

RNA modification, as an epigenetic regulator, has received greater interest in recent years.^34^^,^^35^ m^6^A, as the most common RNA alteration in eukaryotic cells, is critical for carcinogenesis.^36^ A majority of cancers show aberrant expression levels of regulatory proteins associated with m^6^A, which leads to treatment resistance and tumor progression.^37^^,^^38^ In our investigation, we discovered that m^6^A writer METTL16 and reader IGF2BP1 were considerably enhanced in TKI-resistant cell lines. Further research revealed that METTL16 was the methyltransferase of PDK1 mRNA, and IGF2BP1 increased PDK1 levels by increasing the stability of PDK1 mRNA. The results showed that the METTL16/IGF2BP1-mediated m^6^A alteration was the most significant axis for inducing PDK1 stability.

Our investigation found that activated PDK1 has a significant effect on chemoresistance. Thus, targeting PDK1 has potential therapeutic benefit for lung cancer patients, particularly those resistant to TKI treatments. We additionally demonstrated the therapeutic efficacy of the PDK1 inhibitor JX06 in combination with gefitinib, revealing that JX06 induced cell apoptotic rates when combined with gefitinib. Further study using the JC-1 probe revealed that JX06 caused mitochondrial depolarization. Taken together, our findings demonstrated a pivotal role of PDK1 in the TKI acquired resistance; and KDM3A/METTL16/PDK1 is the new axis for mediating TKI resistance as well as a potential therapeutic target for overcoming the resistance.

## CRediT authorship contribution statement

**Zhihao Zhou:** Writing – original draft, Methodology, Investigation, Conceptualization. **Ruike Zhang:** Investigation, Data curation. **Zhaoyang Zhang:** Investigation. **Liyuan Zhang:** Investigation. **Wei Wang:** Investigation, Data curation. **Wenjing Liu:** Investigation, Data curation. **Chunyang Zhang:** Writing – review & editing, Formal analysis, Conceptualization. **Gen Lin:** Data curation, Investigation, Writing – review & editing. **Weimiao Yu:** Writing – review & editing, Methodology, Formal analysis, Conceptualization. **Bo Xu:** Writing – review & editing, Data curation, Conceptualization. **Lin Wang:** Writing – review & editing, Formal analysis, Conceptualization. **Bing-Hua Jiang:** Writing – review & editing, Project administration, Funding acquisition, Formal analysis, Conceptualization.

## Funding

This work was supported in part by the 10.13039/501100001809National Natural Science Foundation of China (No. 82073393), Research and Development Plan of Science and Technology in Henan Province, China (No. 232301420060), Natural Science Foundation of Henan, China (No. 242300421086), and the Science and Technology Research Project of Henan Province, China (No. SBGJ202302023).

## Conflict of interests

Bing-Hua Jiang is the member of Genes & Diseases Editorial Board. To minimize bias, he/she was excluded from all editorial decision-making related to the acceptance of this article for publication. The remaining authors declared no potential conflict of interests.
