# Supplementary material for: PDK1 elevation was induced by epigenetic modifications of KDM3A and METTL16 to mediate TKI resistance and cancer development
Source: Genes Dis. 2025 Nov 25;13(4):101947. doi: 10.1016/j.gendis.2025.101947 (PMC13091346; doi:10.1016/j.gendis.2025.101947)
Supplement: Multimedia component 1 [file mmc1.docx]

## Supplementary Figure legends

**Supplementary Figure 1.** **The PDK1 levels were positively correlated with activation of pyruvate metabolism pathway.** (A-C) Overexpression of PDK1 significantly increased the migration activity, invasion activity and tube-formation activity of PC-9 cells, whereas PDK1 knockdown markedly diminished these activities in PC-9/G cells. (D) The pathway enrichment of TCGA-LUAD cohort was analyzed, and PDK1 levels were positively correlated with carbon metabolism, pyruvate metabolism and glycolysis pathway. (E) The enrichment score (ES) distribution for genes showed that PDK1 levels were positively correlated with pyruvate metabolism pathway.


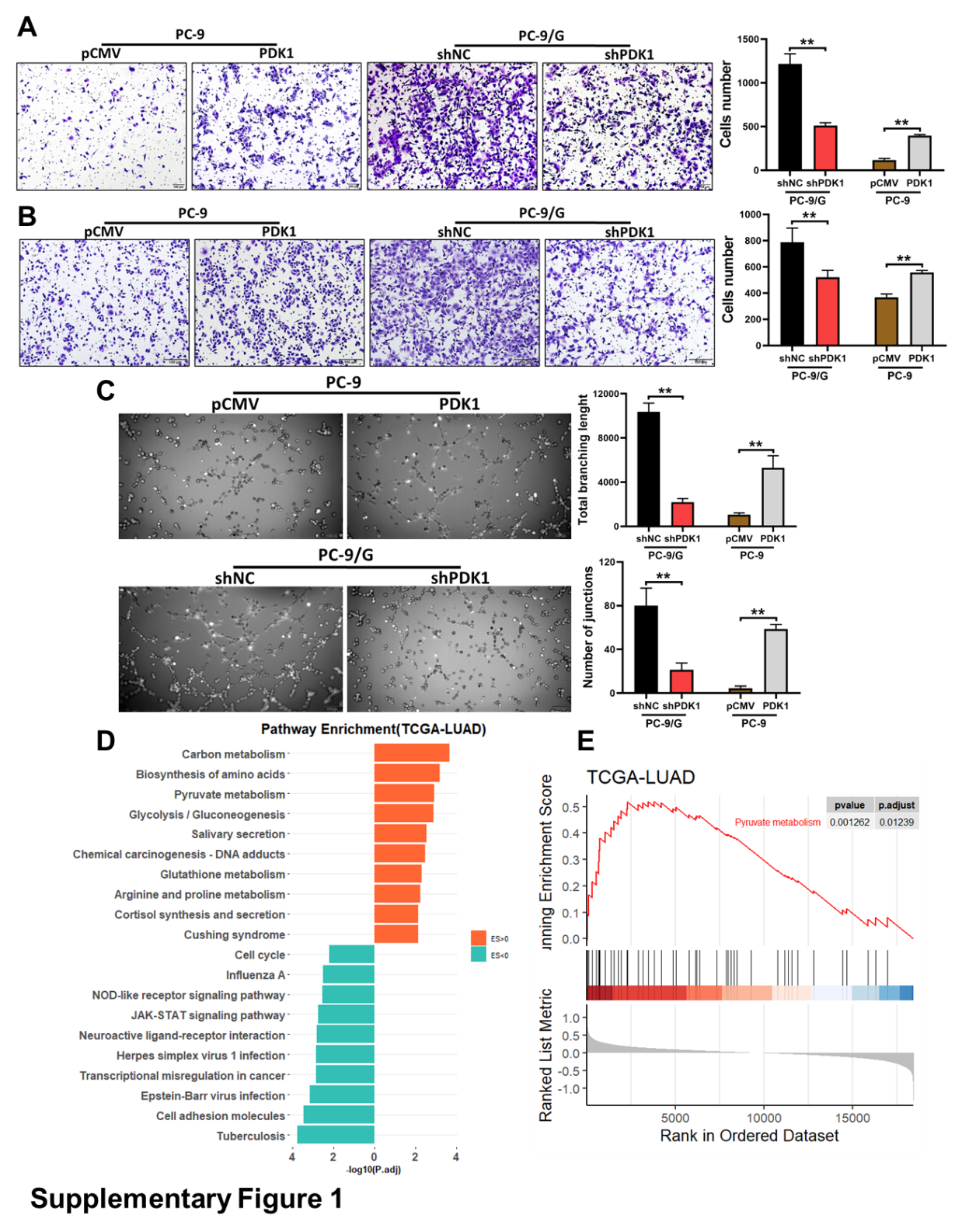


**Supplementary Figure 2.** **PDK1 levels were significantly higher in liver, colon and breast cancer tissues and cells.** (A) Relative PDK1 expression levels in tumor tissues of liver hepatocellular carcinoma, colon adenocarcinoma, and breast invasive carcinoma compared to the normal tissue samples. (B) We measured the relative expression levels of PDK1 in liver cancer cells, colorectal cancer cells, breast cancer cells, and the normal control cells.


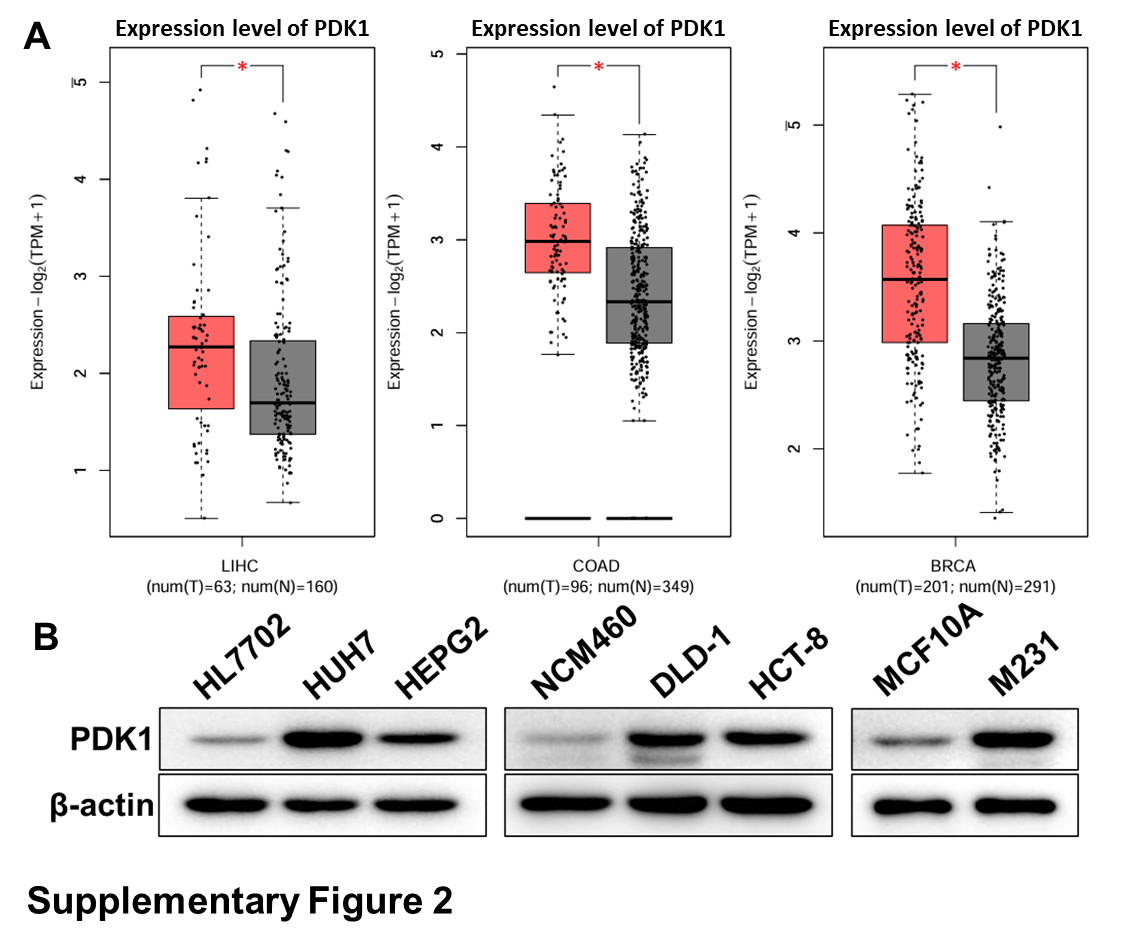


**Supplementary Figure 3. KDM3A regulated cell proliferation, cell migration activities and PDK1 levels.** (A) Relative KDM3A protein levels in tumor and normal samples of lung adenocarcinoma and lung squamous cell carcinoma. (B) Kaplan–Meier overall survival curve was analyzed in lung cancer patients with low (n = 209) and high (n = 304) KDM3A-expressing tumors. (C) We overexpressed KDM3A in PC-9 cells, and silenced KDM3A levels in PC-9/G cells. (D) Overexpression of KDM3A significantly increased the proliferation rates of PC-9 cells, whereas KDM3A knockdown significantly reduced the proliferation rates of PC-9/G cells. (E) KDM3A knockdown attenuated cell migration activities in PC-9/G cells. (F) H3K9me1 and H3K9me2 levels were significantly reduced in PC-9/G cells compared to PC-9 cells. (G) Overexpression of KDM3A in PC-9 cells increased the expression levels of PDK1, and decreased the expression levels of H3K9me1 and H3K9me2. Data were statistically analyzed with Student’s t-test and values are shown as mean ± SD. ** indicates significant difference at p < 0.01.


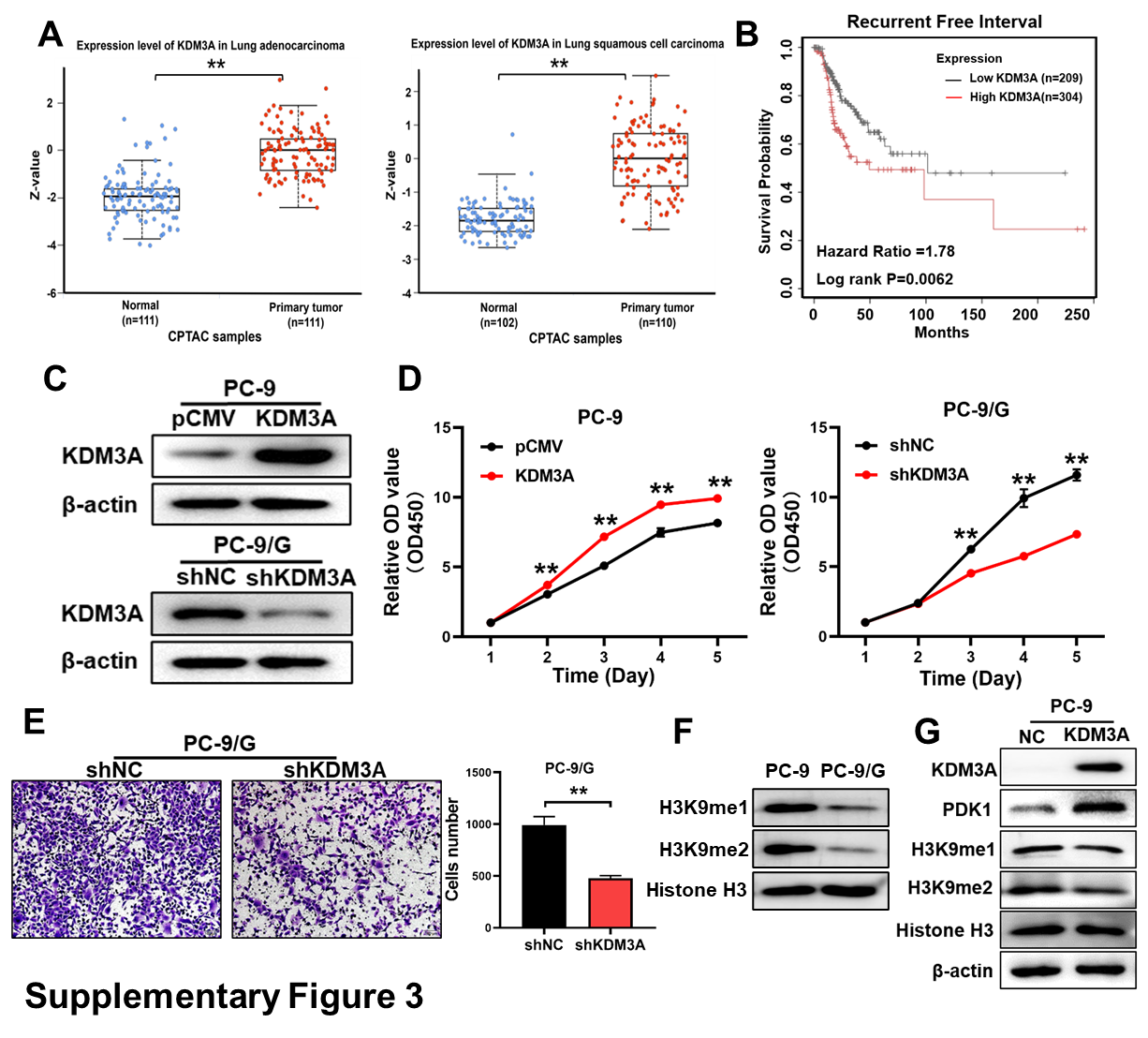


**Supplementary Figure 4.** **METTL16 regulated cell proliferation, cell migration and PDK1 levels.** (A) Among the key m6A methyltransferases analyzed by Western blotting, only METTL16 expression levels were higher in PC-9/G cells than in PC-9 cells. (B) Overexpression of METTL16 induced the m6A levels of PC-9 cells, whereas inhibition of METTL16 decreased the m6A levels of PC-9/G cells. (C) Overexpression of METTL16 significantly increased the proliferation rates of PC-9 cells, whereas inhibition of METTL16 significantly reduced the proliferation rates of PC-9/G cells. (D) METTL16 knockdown attenuated cell migration activities in PC-9/G cells. (E) PDK1 mRNA exhibited a longer half-life in PC-9/G cells (26.76 h) compared to PC-9 cells (9.37 h). (F) PDK1 mRNA levels were determined by semi-PCR in PC-9 cells (control and METTL16 overexpression) after actinomycin D treatment (normalized to 0 hours). Data were statistically analyzed with Student’s t-test and values are shown as mean ± SD. * indicates significant difference at p < 0.05, ** indicates significant difference at p < 0.01.

**
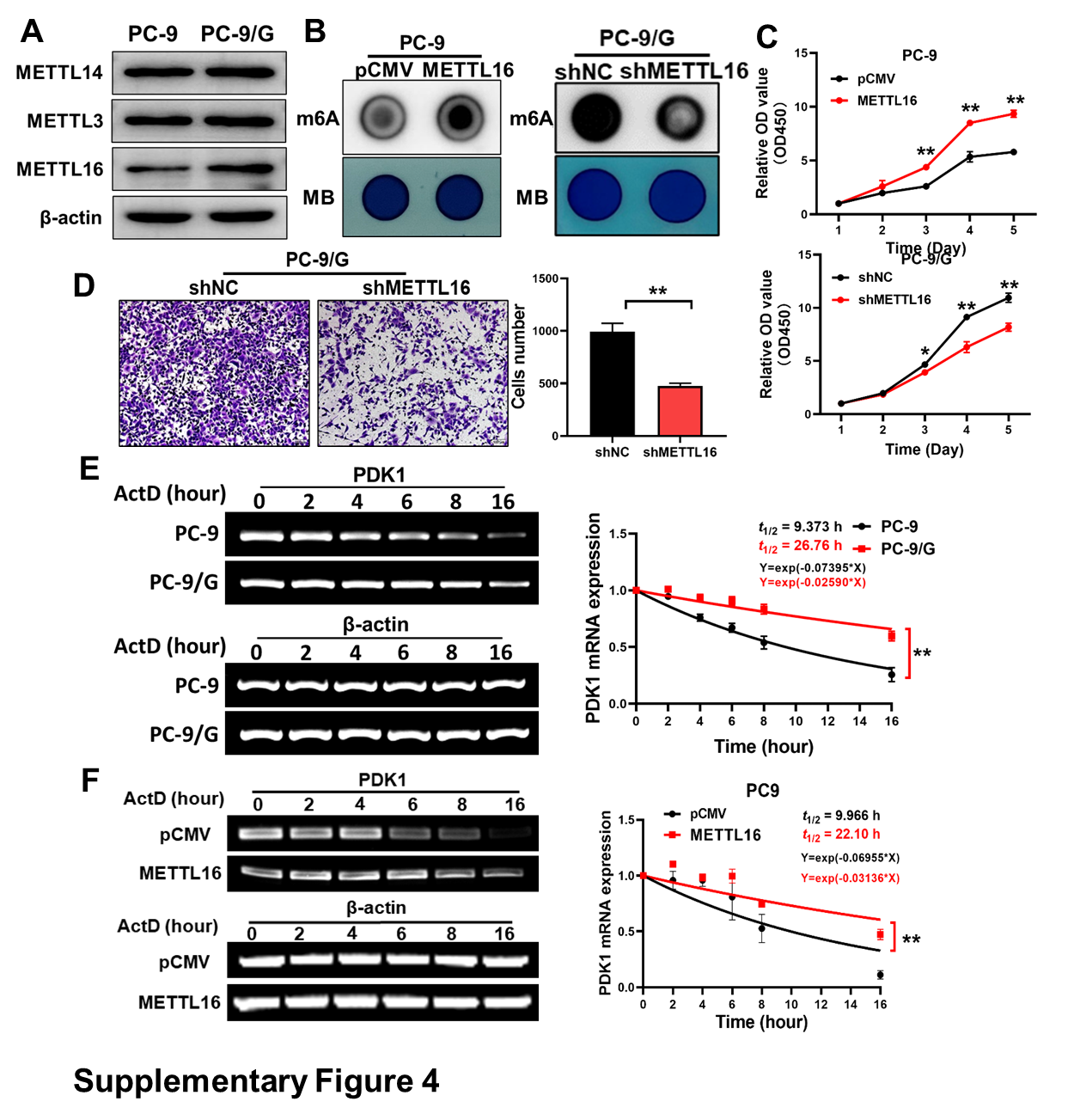
**

**Supplementary Figure 5. KDM3A significantly promoted METTL16 expression levels.** (A) The positive relationship between KDM3A and METTL16 in lung cancer cohort. (B-C) Overexpression of KDM3A significantly increased the expression levels of METTL16 in PC-9 cells, whereas KDM3A knockdown significantly reduced the expression levels of METTL16 in PC-9/G cells. (D) Overexpression of KDM3A induced the m6A levels of PC-9 cells, whereas KDM3A inhibition decreased the m6A levels of PC-9/G cells. (E-F) Co-Immunoprecipitation assay showed that there was an interaction between KDM3A and METTL16. (G) Overexpression of KDM3A wild type plasmid significantly increased METTL16 levels in PC-9 cells, whereas overexpression of KDM3A H1120Y mutant plasmi decreased METTL16 levels.


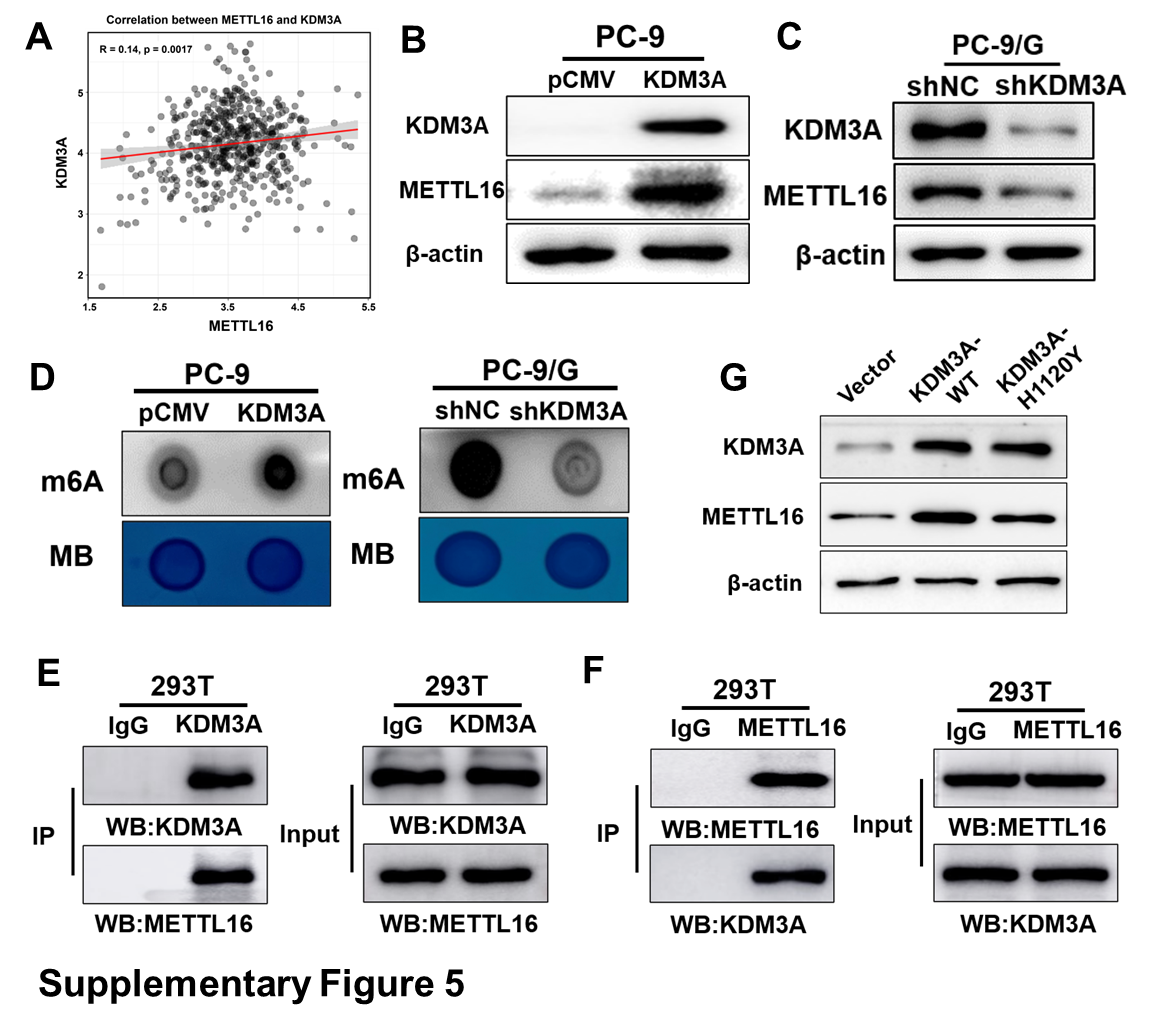


**Supplementary Figure 6. The combination effect of JX06 and gefitinib.** (A) The expression levels of PDK2 and PDK3 did not show significant changes after treatment with PDK1 inhibitor JX06. (B) The combination index (CI) data of JX06 and gefitinib in PC-9/G cells was analyzed.

##
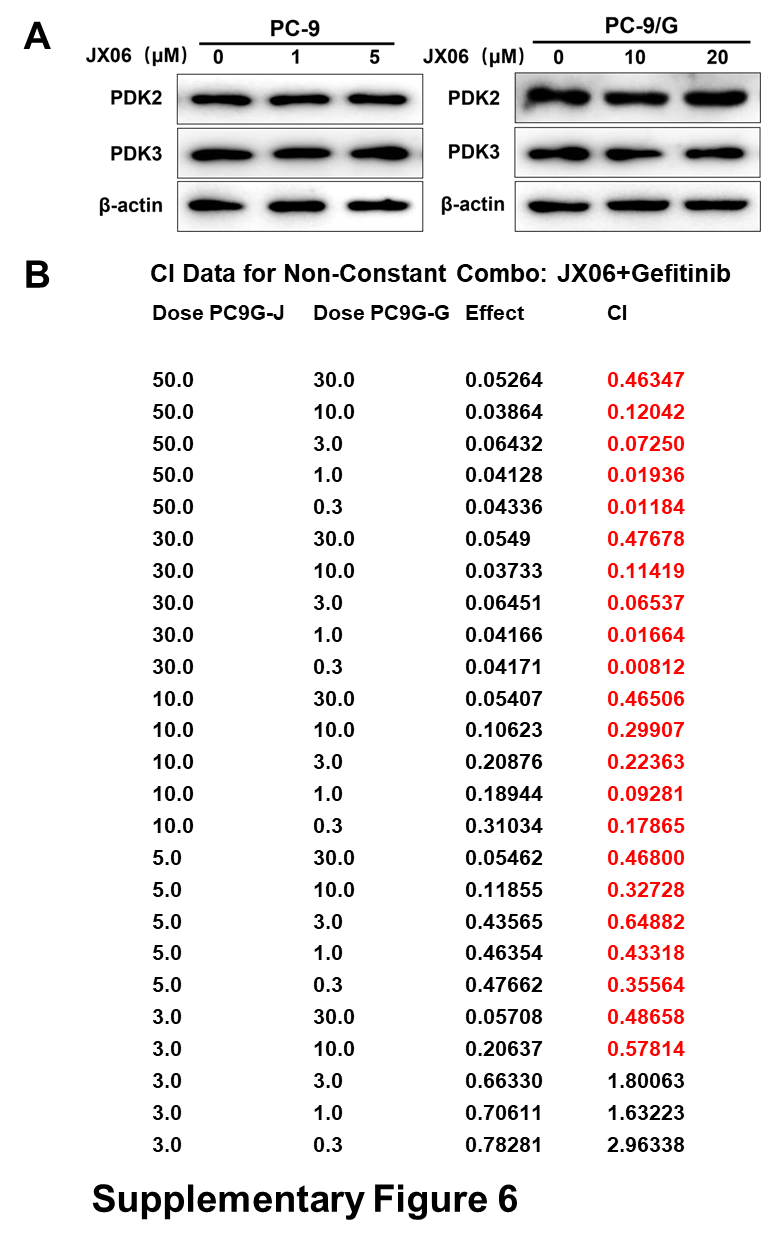


**Supplementary Tables**

**Supplementary Table 1. The primers used for real-time quantitative PCR and semi-PCR**

| Gene | Sequence |
| --- | --- |
| KDM3A | Forward: ACACCGACGTTACCAAGAAGG |
|  | Reverse: CAGGTGACTTTCGTTCAGCTAA |
| METTL16 | Forward: TTCTGTCAAGGTCGGACAATG |
|  | Reverse: CAGCACCACGAATGTTATGGG |
| PDK1 | Forward: AAGCATTACAACACCAACCA |
|  | Reverse: ATTTTCCGATGTCCCAAGT |
| ChIP-PDK1 | Forward: CCTCGGGCACTCAGCAA |
|  | Reverse: CCGTATGGGACATTATTCTTTT |
| Semi-β-actin | Forward: CCTCGCCTTTGCCGATCC |
|  | Reverse: GAGTCCATCACGATGCCAGT |
| Semi-PDK1 | Forward: TGCTGTATGGCCTGCAAGAT |
|  | Reverse: ACCCAGCGTGACATGAACTT |
| RIP-  PDK1 | Forward: AAGCATTACAACACCAACCA |
|  | Reverse: ATTTTCCGATGTCCCAAGT |

**Supplementary Table 2. The antibodies used for western blot or Immunohistochemistry**

| Protein | Company | Catalog Number | Application |
| --- | --- | --- | --- |
| Rabbit polyclonal antibody anti-KDM3A | Proteintech | 12835-1-AP | WB、Co-IP、ChIP |
| Rabbit polyclonal antibody anti- METTL16 | Cell Signaling Technology | 17676 | WB、RIP、ChIP |
| PDK1 | Abcam | 207450 | WB |
| β-actin | Santa Cruz Biotechnology | H1121 | WB |
| Histone H3 Rabbit pAb | ZEN BIO | 381432 | WB |
| Anti-Mono-Methyl-Histone H3 (Lys9) Rabbit pAb | PTMBio | PTM-614 | WB |
| Anti-Di-Methyl-Histone H3 (Lys9) Rabbit pAb | PTMBio | PTM-615 | WB |
| Rabbit polyclonal antibody anti-IGF2BP1 | Proteintech | 22803-1-AP | WB |
| HRP Goat Anti-Mouse IgG | Abcam | AS003 | WB |
| HRP Goat Anti-Rabbit IgG | Invitrogen | 31460 | WB |

**Supplementary Table 3. The primers used for plasmid construction**

| Plasmid | Sequence |
| --- | --- |
| shKDM3A | Forward: CGGATGATCTGATGGCCAACATTCCTCGAGGAATGTTGGCCATCAGATCATTTTTT |
|  | Reverse: ATTAAAAAATGATCTGATGGCCAACATTCCTCGAGGAATGTTGGCCATCAGATCAT |
| shMETTL16 | Forward: CGGCGCAACAGAAGTGGATGATATCTCGAGATATCATCCACTTCTGTTGCGTTTTT |
|  | Reverse: ATTAAAAACGCAACAGAAGTGGATGATATCTCGAGATATCATCCACTTCTGTTGCG |
| shPDK1 | Forward: CGGGCTCTGTCAACAGACTCAATACTCGAGTATTGAGTCTGTTGACAGAGCTTTTT |
|  | Reverse: ATTAAAAAGCTCTGTCAACAGACTCAATACTCGAGTATTGAGTCTGTTGACAGAGC |
| shIGF2BP1 | Forward: CCGGACGCTTAGAGATTGAACATTCCTCGAGGAATGTTCAATCTCTAAGCGTTTTTT |
|  | Reverse: AATTAAAAAACGCTTAGAGATTGAACATTCCTCGAGGAATGTTCAATCTCTAAGCGT |
